# The New Technology of Die Forging of Automotive Connecting Rods from EN AB-71100 Aluminium Alloy Cast Preforms

**DOI:** 10.3390/ma16072856

**Published:** 2023-04-03

**Authors:** Anna Dziubinska

**Affiliations:** Metal Forming and Casting Department, Faculty of Mechanical and Industrial Engineering, Warsaw University of Technology, Pl. Politechniki 1, 00-661 Warsaw, Poland; anna.dziubinska@pw.edu.pl

**Keywords:** die forging, forging presses, automotive connecting rod, forging, cast preform, aluminum alloys, EN AB-71100 alloy

## Abstract

This article presents a new technology for forming automotive connecting rod forgings by means of die forging from cast performs from EN AB-71100 (EN AB-AlZn10Si8Mg) aluminum alloy. A premise was made that the production process would be carried out on forging presses. The process of forming connecting rod forgings was analyzed considering different deformation rates related to the type of machine used: a crank press and a screw press. The billet in the form of in-house designed, shaped preforms cast into sand molds with two variants of geometry was used in the process. The numerical analysis of the new process was carried out on the basis of the finite element method using Deform 3D, the simulation software for metal forming. The simulations were conducted in the spatial deformation conditions, considering the full thermomechanical analysis. Based on the simulations, certain important findings concerning the novel process were acquired, including the distribution of stress, deformation, temperatures, cracking criterion and energy parameters. The results of numerical tests confirmed the possibility of producing defect-free forgings of connecting rods from EN AB-71100 aluminum alloy on forging presses by means of the proposed technology. The proposed process of forging using crank and screw presses was verified in the course of tests conducted in industrial conditions. The properly formed connecting rod forgings were subjected to quality tests in terms of their structure and mechanical properties.

## 1. Introduction

Currently, automotive connecting rods made of hardly deformable aluminum alloys from the 7XXX group (aluminum–zinc–magnesium) are manufactured using die forging, casting and machining [1,2,3].

The best strength-related properties of connecting rods used in the automotive industry are provided by metal forming processes described in the literature [4,5,6,7]. Die forging described in the specialist literature may serve as a perfect example [8,9,10,11,12,13]. This method, however, imposes certain limitations, as the production of connecting rods from less ductile 7XXX aluminum alloys is difficult. In the case of die forging of connecting rods from the aluminum–zinc–magnesium group, a cylindrical, plastically formed billet is used, usually extruded or in the form of rolled preforms [9,14,15]. The process is carried out in several stages with a large allowance for flash. Approximately 50% of the mass of the forging constitutes a technological waste [16,17]. The process uses a special die impression design compared to processing other, more ductile aluminum alloys. Additionally, a lower degree of one-time deformation is applied due to the occurrence of cracking. For die forging of automotive connecting rods from the 7XXX alloy group, additional auxiliary dies for initial forging must be made. This method of die-forging of automotive connecting rods from less ductile aluminum alloys is characterized by high consumption of material, labor and energy and low process efficiency.

Automotive connecting rods manufactured by the casting technology have significantly lower mechanical and functional properties compared to elements produced by metal forming methods presented in the literature [11,18,19,20]. Cast automotive connecting rods have casting defects, which include heterogeneity of structure, coarseness, bubbles, porosity, shrinkage cavities or thinning, which affect their properties [21,22].

In the production of automotive connecting rods, the machining technology described in the literature is used [23,24,25]. The machining of connecting rods gives the surfaces the desired shape, dimensions and surface quality by removing material from the charge in the form of a cuboid or cylinder using cutting tools. This technology is characterized by high labor, time and energy consumption of the process and generation of large material loss as well as low-quality processed products.

Despite the existence of these technologies, new solutions are still being sought. The main objective in developing the new technology was to reduce the number of operations required to obtain a connecting rod compared to the currently used multistage forging of connecting rod forgings and to obtain improved product properties compared to the currently used casting machining technology. The current article presents a novel technology for forming automotive connecting rod forgings by die forging from preforms cast from EN AB-71100 aluminum alloy. In the new technology of forging an automotive connecting rod forging, the geometry of the cast preform will be similar in shape to the forging, particularly in terms of the outline in the die parting plane. The new process will be carried out in a single forging operation in the finishing impression using typical forging presses (crank and screw presses) and with the application of inexpensive methods of heating the tools (furnace, gas burner). The proposed technology is unique in the world. Its basic principles were granted patent protection.

## 2. Research Methodology

### 2.1. Assumptions of the New Technology, Material and Numerical Simulations

The research concerned the novel process of forging an automotive connecting rod forging made of a billet in the form of a shaped preform made of EN AB-71100 aluminum alloy cast in sand molds [26,27]. The chemical composition of the test material used for preforms is shown in Table 1.

**Figure 1:**
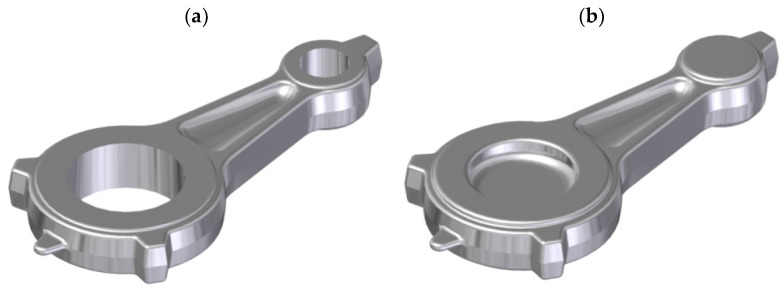
Three-dimensional model of the finished connecting rod part (**a**) and the designed connecting rod forging (**b**).

**Figure 2:**
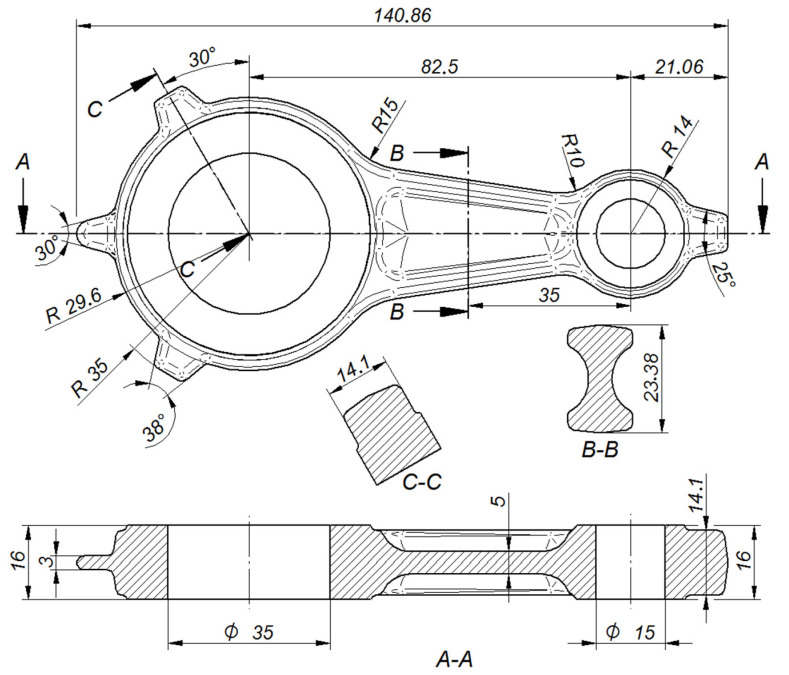
Selected dimensions of the finished connecting rod.

**Figure 3:**
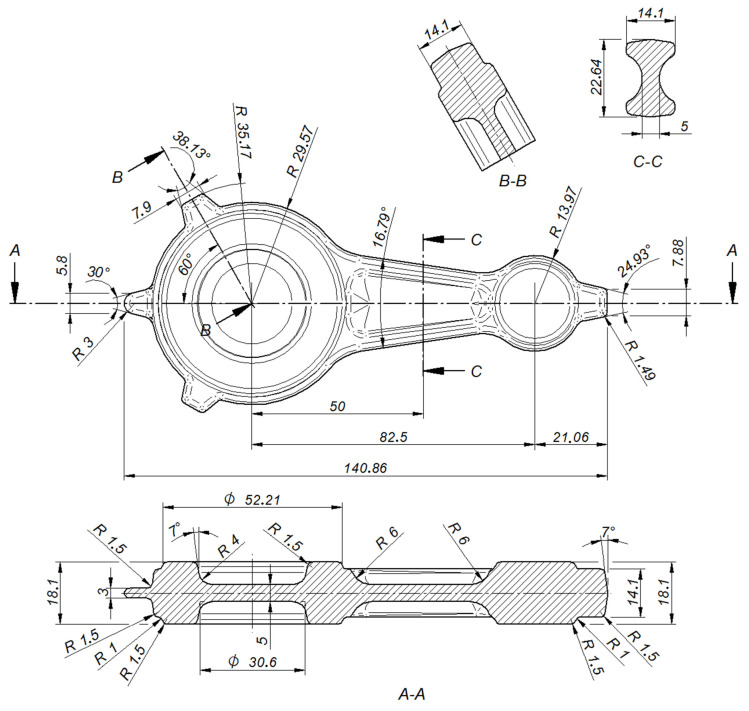
Selected dimensions of the connecting rod forging. The design of the forging process for the forging of the connecting rod from the cast preform commenced with the drawing of the cross-sectional diagram of the forging shown in Figure 4. The horizontal axis labeled “length of forging/preform [mm]” presents the distance of individual cross-sections of the forging/preform from the origin of the coordinate system. The vertical axis labeled cross-sectional area (mm^2^) presents the size of the forging/preform cross-sectional area. Two preforms (variants 1 and 2) were designed based on the cross-sections of the forging, the outline of the forging in the die parting plane, and by changing the characteristic dimensions of the forging (see Figure 5, Figure 6, Figure 7 and Figure 8). The cross-sectional areas of the designed preforms are compared with the forging in Figure 4. The designed preforms differ primarily in height and thus in the degree of forging, cross-sectional dimensions in the die parting plane and volume. For variants 1 and 2, the degree of forging defined by the ratio of the height of the preforms to the height of the forging is h_1_/h_0_ = 1.93 and h_2_/h_0_ = 1.43, respectively (Figure 5). The volume of variant 1 preform shown in Figure 6a is 67,437.2 mm^3^ (the mass is 0.192 kg), while that of variant 2 preform shown in Figure 6b is 71,743.6 mm^3^ (the mass is 0.204 kg). As assumed, the forging of the connecting rod forging from the cast preform will take place only in the finishing impression of the dies.

The automotive connecting rod shown in Figure 1a and Figure 2 was selected for the tests. Based on the geometrical model and executive drawing of the connecting rod, a forging was designed, and the 3D model and its geometry were developed (see Figure 1b and Figure 3). A web was added to the hole in the connecting rod foot while the hole in the connecting rod head was plugged. On the side surfaces adjacent to the connecting rod holes, material allowances were added to allow the final machining. The remaining surfaces of the forging were left unchanged in relation to the finished product. The volume of the finished part (Figure 1a) is 44,610.3 mm^3^ (the mass is 0.127 kg), while that of the designed forging (Figure 1b) is 58,299.9 mm^3^ (the mass is 0.166 kg). The material waste in the form of machining allowances is approx. 13,689.6 mm^3^ (i.e., the mass is 0.028 kg).

Tests of the connecting rod die forging process from the designed preforms were carried out based on the FEM analysis in the Deform 3D software (v. 11, Scientific Forming Technologies Corporation, Columbus, OH, USA) [30]. The numerical simulations of the studied processes were conducted based on the assumption of spatial deformation and the application of the full thermomechanical model. The preforms and dies were designed in the Solid Edge software (v. ST10; Siemens Digital Industries Software, Plano, TX, USA).and subsequently imported into the preprocessor module of Deform. The billet in the form of variant 1 and variant 2 preforms was modeled as a metal-forming element divided into 150 thousand tetragonal elements. The dies were modeled as rigid bodies. The simulations employed AB-71100 aluminum alloy selected in the course of research. The material model of the above-mentioned alloy in the form of casting in sand molds was developed on the basis of its own plastometric tests carried out on the DIL 805A/D deformation dilatometer(BAHR-Thermoanalyse, Hüllhorst, Germany). Flow stresses were entered into the software in a tabular form and depended on the temperature in the 400–500 °C range, strain rate from 0.01 s^−1^ to 10 s^−1^ and strain deformation values in the 0–1 range. The calculations were based upon the premise that the billet is heated up to 480 °C, and the temperature of the processing tools amounts to 250 °C. For the crank press, the movement in the Deform software was defined in line with the machine’s parameters, i.e., crank press; additionally, the total stroke of 15 mm and press cycle of 12 min^−1^ were given. For the screw press, the movement in the Deform software was defined in line with the machine’s parameters, i.e., screw press; additionally, the impact energy of 40 kJ, a moment of inertia of moving elements at 557.8 kgm^2^ and a thread lead of 188 mm were given. The friction conditions between the billet and the tools were described using the constant friction law assuming the friction coefficient m = 0.15 [31]. The assumed heat transfer coefficient between the above objects was equal to 7.45 kW/m^2^K, while between the billet and the environment, the coefficient was equal to 0.02 kW/m^2^K.

### 2.2. The Conditions for the Experimental Tests for the Forging of the Connecting Rod Forgings

**Figure 9:**
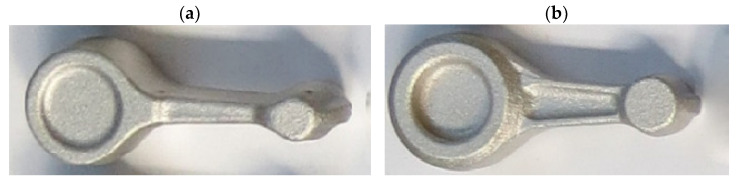
Pictures of connecting rod preforms: (**a**) variant 1; (**b**) variant 2.

Preforms made by casting into sand molds from AB-71100 aluminum alloy were used in the experiments. On the basis of numerical tests, two geometries of preforms were developed, with a greater degree of forging (variant 1) (Figure 9a) and a lower degree of forging (variant 2) (Figure 9b), with geometries similar in the outline to the geometry of the forging in the die parting plane.

**Figure 10:**
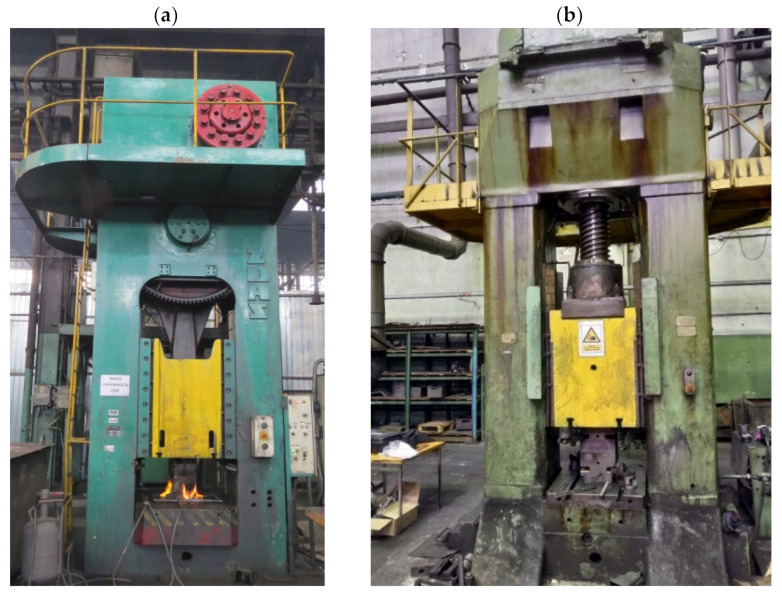
Pictures of forging presses used for tests in industrial conditions: (**a**) ZDAS LU 400/1000 crank press; (**b**) F1736A screw press.

**Figure 11:**
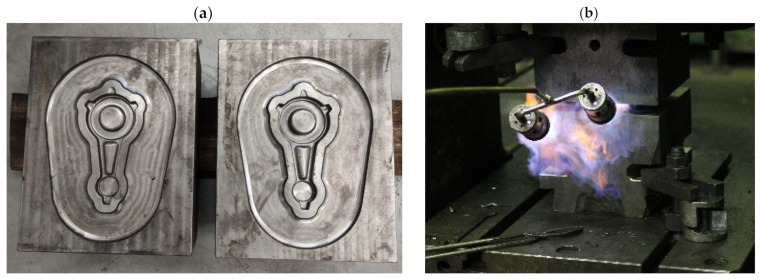
(**a**) Dies used for tests with finishing impressions (**b**) and the method of maintaining die temperature using gas burners.

**Figure 12:**
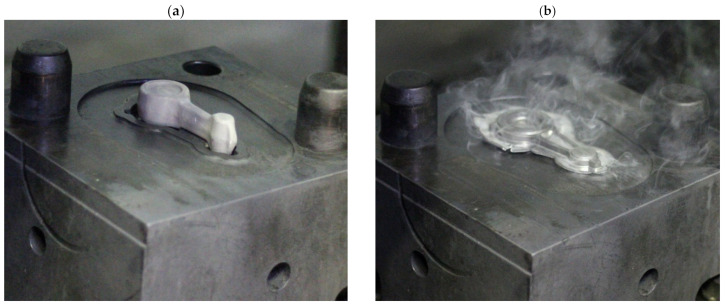
An example of a connecting rod preform (variant 1) placed in the finishing impression (beginning of the process) (**a**) and forging formed on the press (end of the process) (**b**).

Prior to die forging, all preform castings were subjected to the annealing process using furnace heating to the temperature of T = 480 °C, then holding at the temperature of T = 480 °C for 24 h and cooling in a furnace to room temperature (Table 2). These conditions were developed based on the review of the literature, standards and own research [32,33,34]. Experimental tests were conducted on presses in industrial conditions. The forging processes were carried out on ZDAS LU 400/1000 crank press (ZDAS, Zdar, Czech Republic) (Figure 10a) and F1736A screw press (Stanko, Rostov-on-Don, Russia) (Figure 10b). The dies for the tests were heated in a furnace to 250 °C. During the tests, their temperature was maintained using gas burners (Figure 11). The preforms were heated to a forging temperature of 480 °C within 35 min. Then, the preforms were forged in a single operation in the finishing impression (Figure 12).

After forging on each of the machines (crank press, screw press), the forgings were trimmed of flash and etched in five baths with the following parameters [17]:

- I bath—warm water, 50–70 °C;
- II bath—cold water;
- III bath—nitric acid aqueous solution HNO3 (approx. 20–35%);
- IV bath—cold water;
- V bath—sodium hydroxide aqueous solution NaOH (approx. 10%) 50–70 °C.

Subsequently, heat treatment was conducted in the temperature conditions presented in Table 2.

### 2.3. Types of Qualitative Tests Conducted for the Formed Forgings of the Connector Rod

In order to assess the quality of the connecting rod forgings made of AB-71100 aluminum alloy obtained during industrial trials, tests of the structure and mechanical properties were carried out.

**Figure 13:**
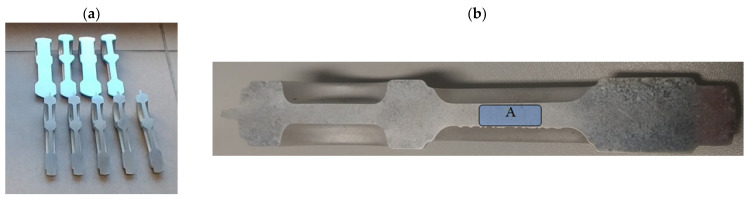
Analyzed cross-sections of connecting rods during preparation (**a**) and the tested area of preforms and forgings (**b**).

Microstructural tests were conducted on longitudinal sections of castings (preforms) and connecting rod forgings (Figure 13).

The preparation of metallographic specimens was carried out by grinding the surface of the cross-sections using abrasive discs with grain sizes of 80, 120, 240, 320, 600, 800 and 1200. Subsequently, the specimens were polished using diamond slurries with grain sizes of 3 μm and 0.05 μm. The surfaces were etched with Keller’s reagent (1 mL HF, 2.5 mL HNO3, 1.5 mL HCL, 95 mL H2O) by immersion for approx. 30 s. Microstructural tests were performed using a NIKON MA200 optical microscope (Tokyo, Japan). Hardness measurements were carried out using the Vickers method with the Future-tech FM800 hardness tester (Future-Tech Corporation, Kawasaki, Japan). The measurements were made on the HV 0.5 scale in accordance with the PN-EN ISO 6507-1:2006 standard.

**Figure 14:**
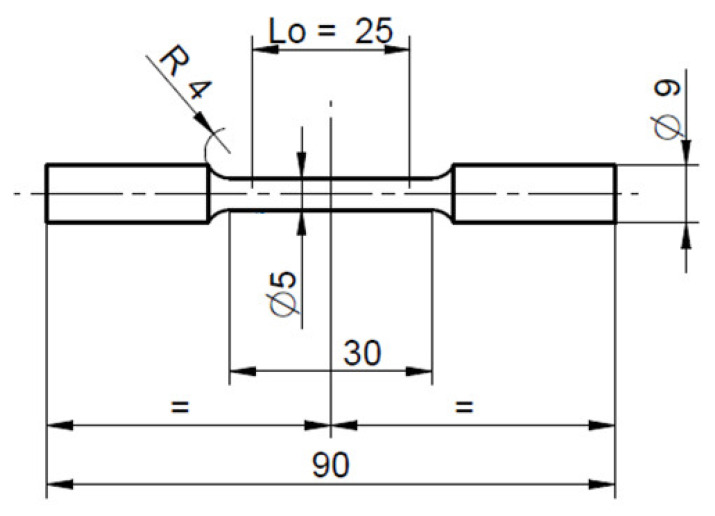
Dimensions of specimens for tensile testing prepared from preforms and forgings.

**Figure 15:**
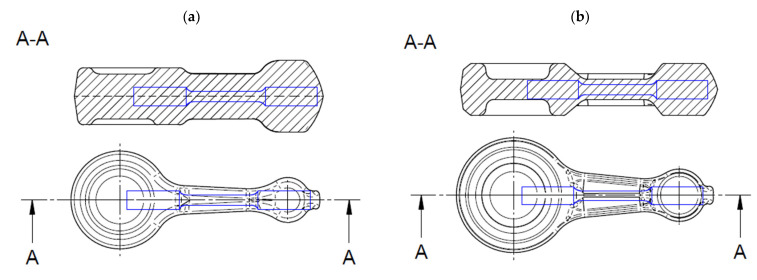

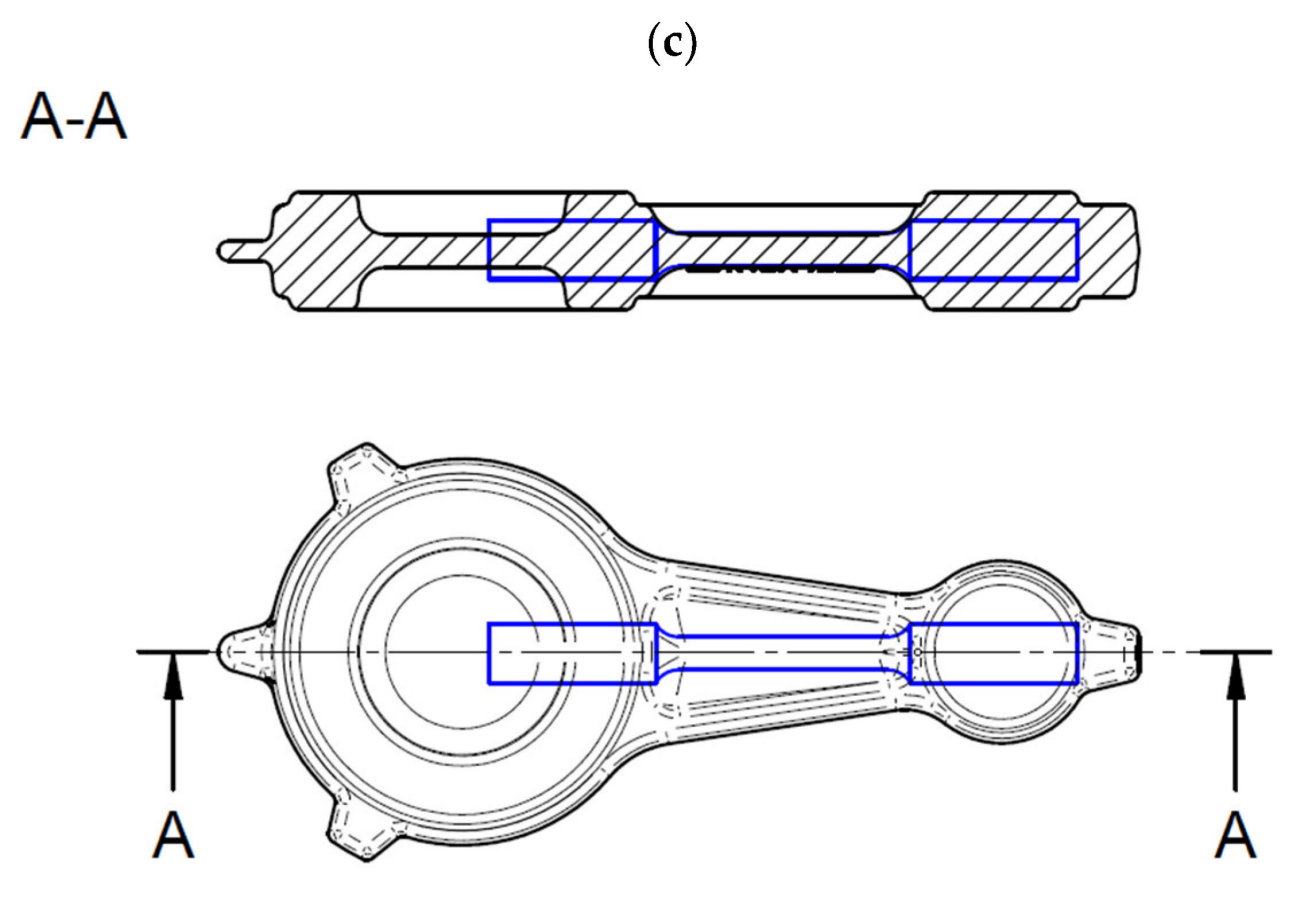
Sampling locations for mechanical properties testing marked with a blue line: (**a**) variant 1 preform; (**b**) variant 2 preform; (**c**) forging.

**Figure 16:**
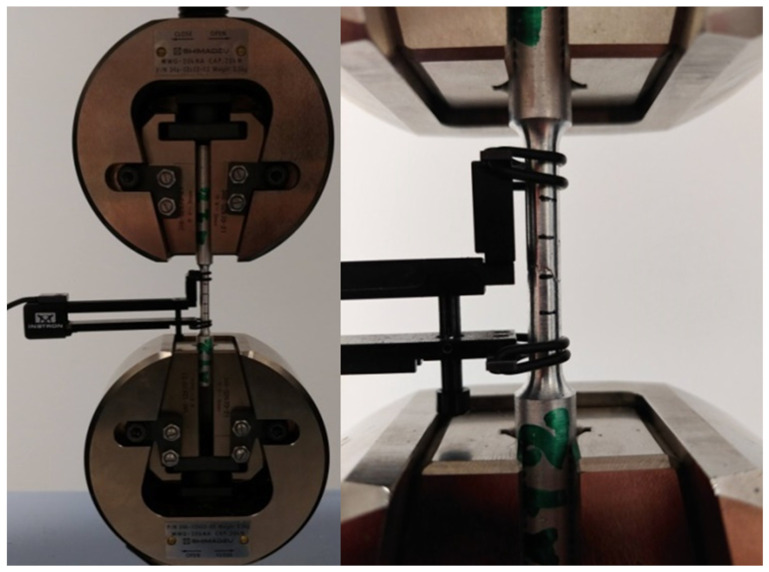
A sample made of connecting rod forgings placed in a testing machine.

Mechanical tests were carried out on samples made of connecting rod shanks). The dimensions of the specimens for tensile testing and the sampling locations for testing mechanical properties are shown in Figure 14, Figure 15 and Figure 16. The geometry of the samples and the test procedure were applied in accordance with ISO 6892-1 standard. A Shimadzu AG-X plus 20KN machine (Shimadzu Corporation, Kyoto, Japan) was used for the tests, equipped with a longitudinal extensometer to measure deformations and control the test speed.

## 3. Results and Discussion

### 3.1. Results of Numerical Simulations

**Figure 17:**
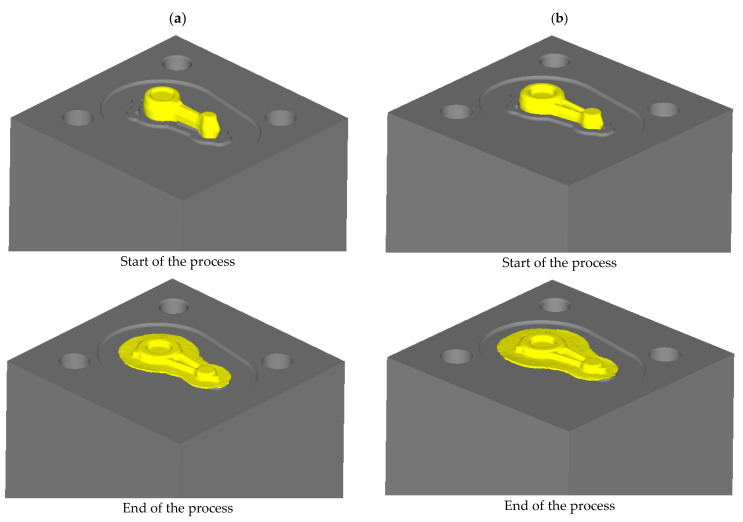
Scheme of the forging process of the connecting rod forging from the preform according to variants: (**a**) 1; (**b**) 2 (top tool was removed to facilitate the illustration of the process).

Figure 17 shows a diagram of the connecting rod forging process extracted from the FEM simulation. In both variants, the impression was completely filled with material, the excess of which was transformed into flash. When designing the die-forging process, it is necessary to ensure that the flash is not overly large and that it is even along the forging outline in the die-parting plane, which was achieved in the proposed technology.

**Figure 18:**
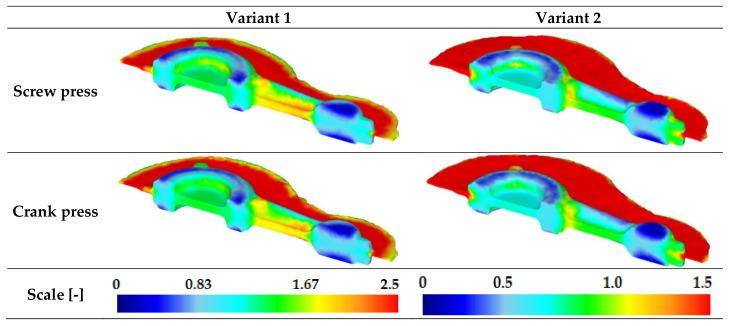
Distribution of effective strain for connecting rod forgings made of AB-71100 aluminum alloy formed based on the new technology.

Figure 18 shows the effective strain distribution for connecting rod forgings made of AB-71100 alloy formed following the novel technology. For forgings forged from preforms in line with variants 1 and 2, the maximum values of effective strain are up to 2.5 and 1.5, respectively. With regard to a given variant of the preform, it was observed that the type of forging machine does not significantly affect the distribution of effective strain in terms of quantity and quality.

**Figure 19:**
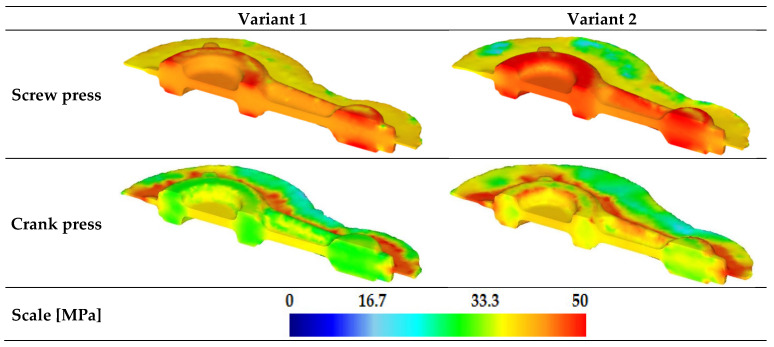
Distribution of effective stress for connecting rod forgings made of AB-71100 aluminum alloy formed based on the new technology.

The analysis of effective stress distribution showed that for forgings made of AB-71100 aluminum alloy, the maximum values of the discussed parameter reach 50 MPa (Figure 19). Stress values are similar in the entire volume of individual forgings for a given machine. It was noted that for the crank press, the highest stress values in the forging occur in the area of the flash land, and in the case of the screw press, they are located in the area of the forging geometry. The maximum value of 50 MPa in the forgings is low and should affect neither the homogeneity of the specific area nor the durability of the die imprint in the areas. The analyzed variants of the forging geometry do not significantly affect the stress distribution in the forging itself.

**Figure 20:**
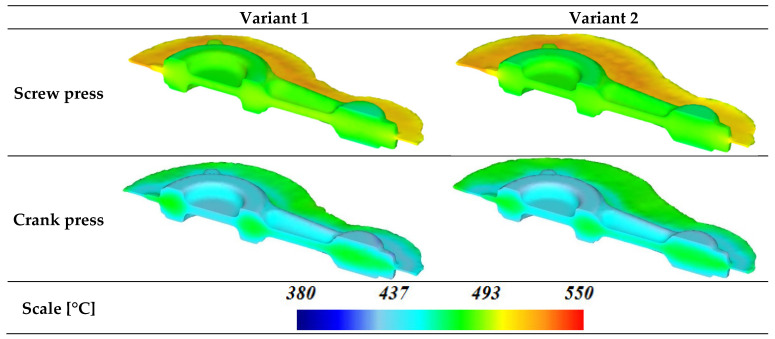
Temperature distribution for connecting rod forgings made of AB-71100 alloy formed based on the new technology.

On the basis of the analysis of temperature distribution in the connecting rod forgings formed following the proposed technology, it was detected that the type of forging machine used has the greatest influence on the temperature (Figure 20). This translates directly into the rate of deformation and the timing of the process. Thus, the forging time is shorter for the screw press and longer for the crank press. As a consequence, the temperature values are the lowest in forgings forged on a crank press. In this case, the temperature values drop to 430 °C from the initial value of 480 °C. Temperature drops are the greatest in the areas of contact between the material and the dies. In the case of forging on the screw press, temperature values oscillate around the initial material temperature of 480 °C in the area of the connecting rod forging and are slightly higher in the area of the flash and amount to 500 °C.

Along with the analysis of the above-mentioned parameters, the risk of crack formation in the forging was studied. For the theoretical analysis of this phenomenon, the Cockroft–Latham (C-L) failure criterion in a modified form was input in the Deform software [16,38]. The software determines locations at the risk of cracking based on this criterion expressed by the following formula:

(1) $$\int_{0}^{\varepsilon_{p}}\frac{\sigma_{\max}}{\sigma_{H}}d\varepsilon =C_{1}$$

where σ_max_ is the maximum principal stress, σ*_H_* is the equivalent stress according to Huber’s hypothesis, *ε* is the strain intensity, and *C*_1_ is the integral value.

The C-L criterion assumes that when the work performed by tensile stresses in uniform tension reaches a certain critical value *C*_1_ = CCL, plastic fracture of the material will occur.

**Figure 21:**
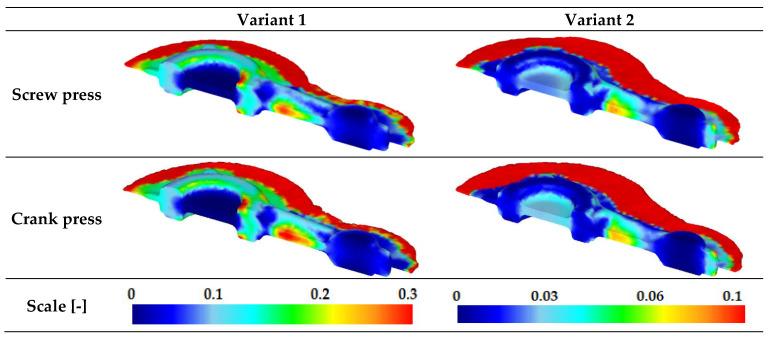
Distribution of Cockcroft–Latham integral values for connecting rod forgings made of AB-71100 alloy formed based on the new technology.

Based on the analysis of the distribution of Cockcroft–Latham integral values (Figure 21), it was found that the distribution of this parameter in terms of quality is similar in all analyzed cases. The highest values are located in the shank part of the connecting rod forging and the smallest in the head of the connecting rod. It was observed that the geometry of the preform has the greatest influence on the values of the integral. Within the forging, the values of the integral for variant 1 are up to 0.3, and for variant 2, up to 0.1. Therefore, variant 2 preform, characterized by a lower degree of forging, seems to be safer in terms of the possibility of the material’s loss of cohesion. Nevertheless, values up to 0.3 appearing for variant 1 of the preform seem to be safe for hot forging of aluminum alloys.

**Figure 22:**
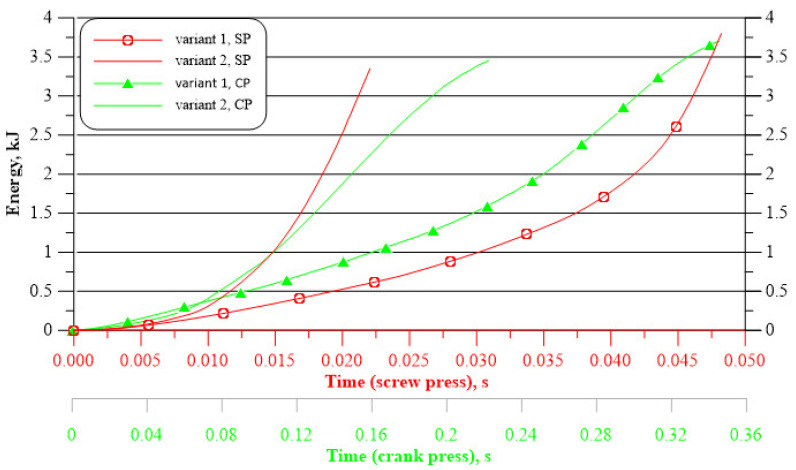
Impact energy of the upper die as a function of time for forging a connecting rod forging from preforms made of 71100 alloy.

For further comparative analysis of the technology of forging connecting rods f from cast preforms, diagrams of the impact energy of the upper die as a function of time for individual preforms and forging presses were presented (Figure 22). For individual preforms, it was noted that, regardless of the forging machine, the energy consumption of the processes is similar. Larger differences are visible between the geometrical variants of the preforms. Forging a connecting rod from variant 1 preform requires the use of more energy (3.80 kJ for the screw press; 3.70 kJ for the crank press) than in the case of forging from variant 2 preform (3.35 kJ for the screw press; 3.45 kJ for the crank press). Based on the diagrams of the impact energy of the upper die as a function of time for individual preforms and forging presses, the efficiency of the analyzed processes was determined in view of the forging time for the individual presses. For two geometries, the process is more efficient on the screw press (variant 1 preform, forging time amounts to 0.048 s, variant 2 preform, forging time equal 0.022 s). On the other hand, the process is less efficient on the screw press (variant 1 preform, forging time equal 0.34 s, variant 2 preform, forging time equal 0.22 s).

A comparative analysis of the new technology of forging from cast preforms and the technology of multistage forging from a bar of diameter Ø30 mm and length 148 mm (one currently used in the industry) was carried out on the basis of the material consumption of the process. Table 3 summarizes the volume of geometry for each forging variant and the percentage of process waste for each technology.

Based on the numerical analyses, the following conclusions were formulated:

- On the basis of numerical simulations, it was found that in the proposed forging process, forgings of connecting rods made of AB-71100 aluminum alloy can be obtained from cast preforms;
- Both geometric variants of preforms ensure that forging of the assumed shape and dimensions is obtained;
- Forging from a preform with a lower degree of forging (variant 2) seems to be a safer option in terms of the possibility of losing the cohesion of the material; this is supported by lower values of the Cockcroft–Latham integral;
- Forming a forging from variant 2 preform is characterized by lower energy consumption than in the case of variant 1;
- Due to the final temperature of the forging, it seems more advantageous to forge on a screw press than on a crank press;
- Forging of the connecting rod forging from the cast preform is carried out in a single step exclusively, which shortens production time in relation to the currently applied multistage technologies of their production.

### 3.2. Results of Experimental Tests

#### 3.2.1. Results of Experimental Tests of Forging Connecting Rod Forgings from Cast Preforms

**Figure 23:**
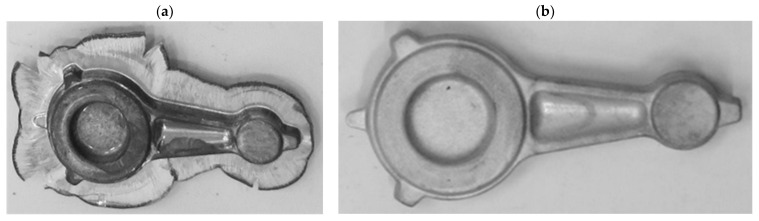
Forgings of connecting rods made of AB-71100 aluminum alloy obtained in the process of forging on a screw press from a cast preform in accordance with variant 1 (**a**); trimming the flash and etching after the process (**b**).

**Figure 24:**
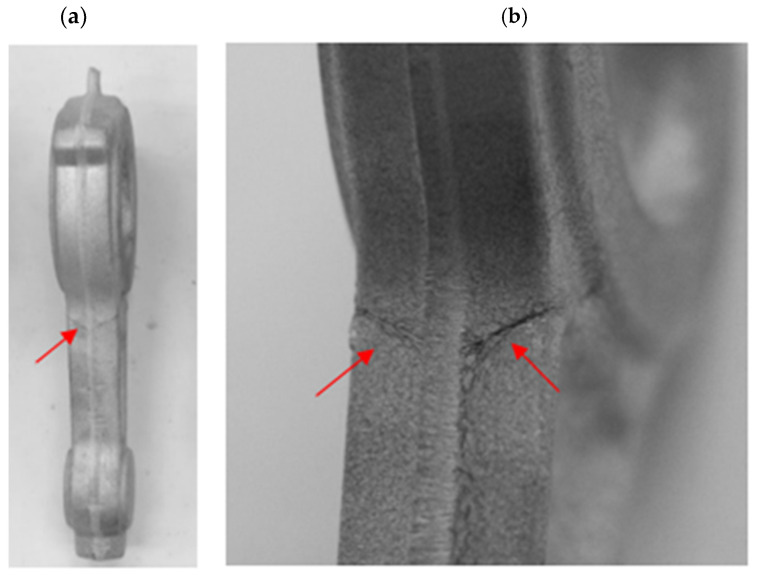
Forgings of connecting rods made of AB-71100 aluminum alloy after trimming the flash and the etching process, obtained from a preform in accordance with variant 1 with visible cracks (**a**); detailed view of the location of cracks in the forging (**b**).

On the basis of the experimental tests, it can be observed that during the forging process of connecting rods forgings from preforms made of AB-71100 aluminum alloy on the screw press in accordance with variant 1, no defect-free products were obtained (Figure 23 and Figure 24). The defective forgings had cracks located in the part of the shank near the head of the connecting rod (Figure 24a,b). It should be noted that no cracks occur in the simulation results for this case. This stems from idealized simulation conditions as well as the higher rigidity of the billet when divided into much larger pieces than the particles of the actual processed material. Additionally, the emergence of cracks during the initial stage of the process may have been concealed by remeshing at a later stage. As a consequence, defects such as cracking may only be revealed during experimental tests.

**Figure 25:**
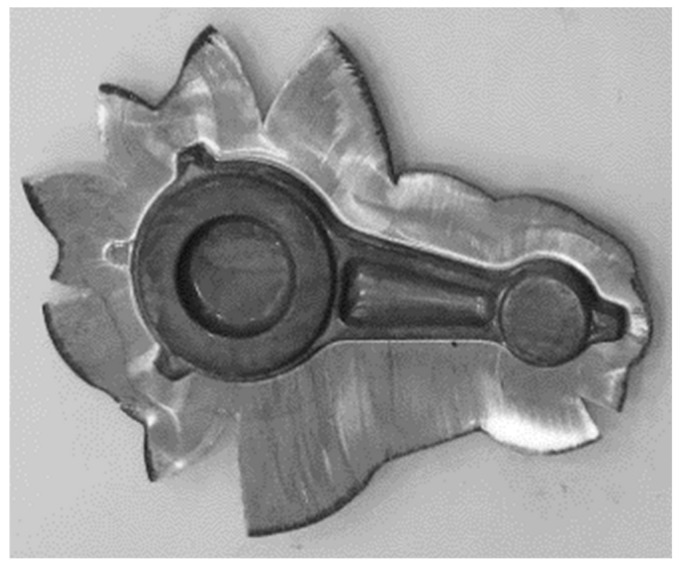
Forgings of connecting rods made of AB-71100 aluminum alloy obtained in the process of forging on a screw press from cast preforms in accordance with variant 2.

**Figure 26:**
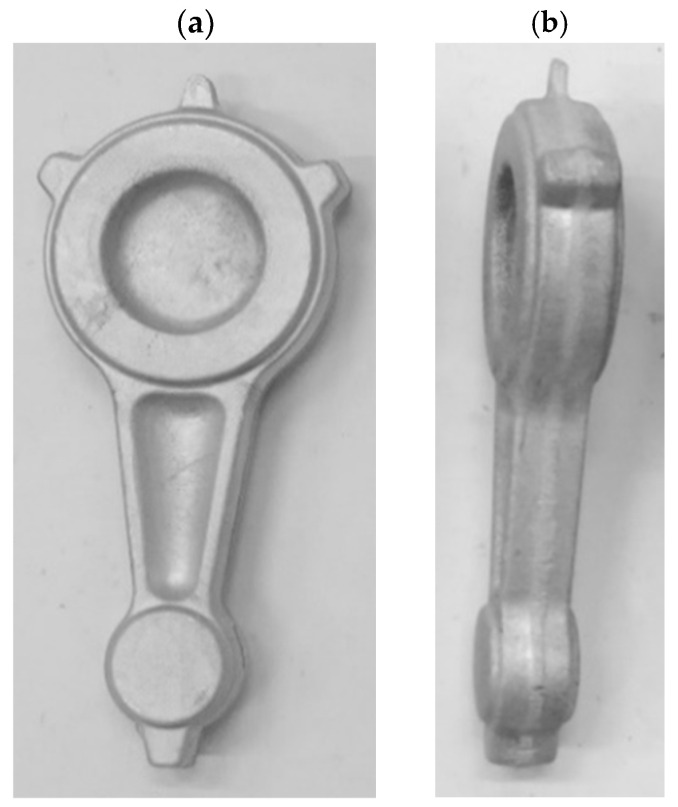
Connecting rod forgings made of AB-71100 aluminum alloy after flash trimming and etching, obtained from cast preforms in accordance with variant 2: (**a**) top view; (**b**) side view.

During the process of forging the connecting rod forgings from variant 2 preform on a screw press, defect-free forgings were obtained (Figure 25 and Figure 26). The formed forgings were subjected to the process of trimming the flash and etching. The final forgings without flash are shown in Figure 26.

**Figure 27:**
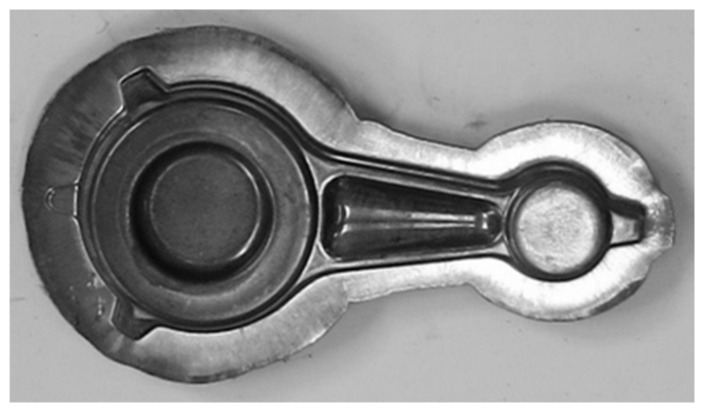
Forgings of connecting rods made of AB-71100 aluminum alloy obtained in the forging process on a crank press from cast preforms in accordance with variant 1.

**Figure 28:**
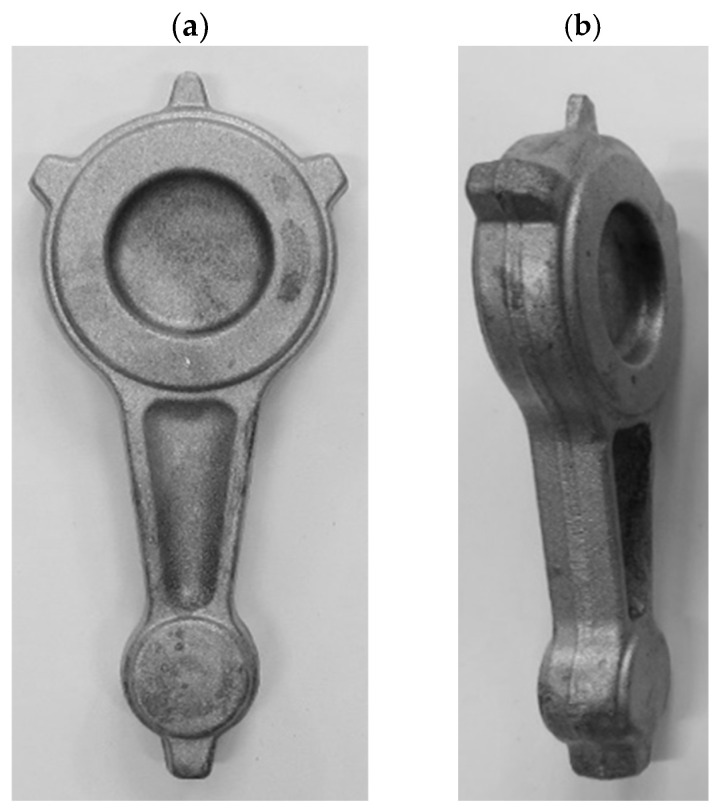
Forgings of connecting rods made of AB-71100 aluminum alloy after trimming the flash and etching process obtained from cast preforms in accordance with variant 1: (**a**) top view; (**b**) side view.

The tests of the forging process performed on a crank press for the forging of connecting rods from preforms made of AB-71100 aluminum alloy in accordance with variant 1 made it possible to obtain defect-free products (Figure 27 and Figure 28). The formed forgings were subjected to the process of trimming the flash and etching. The final forgings are shown in Figure 28.

**Figure 29:**
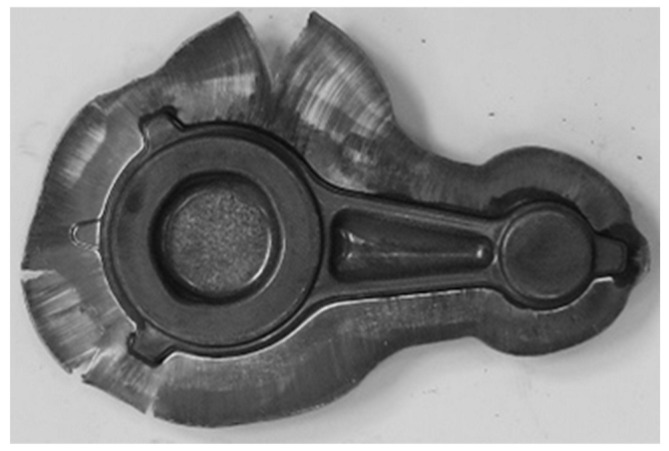
Forgings of connecting rods made of AB-71100 aluminum alloy obtained in the process of forging on a crank press from cast preforms in accordance with variant 2.

**Figure 30:**
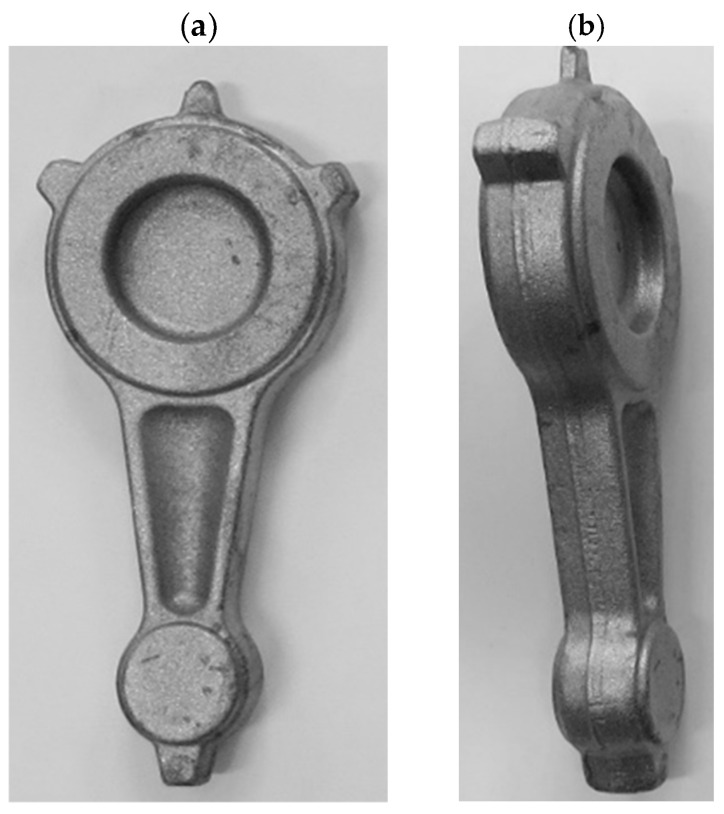
Forgings of connecting rods made of AB-71100 aluminum alloy after the process of trimming the flash and etching obtained from cast preforms in accordance with variant 2: (**a**) top view; (**b**) side view.

The process of forging connecting rod forgings from preforms in accordance with variant 2 on a crank press also resulted in the formation of defect-free forgings (Figure 29 and Figure 30). The obtained forgings were subjected to the process of flash trimming and etching. The final connecting rod forgings are shown in Figure 30.

When analyzing the test results, it can be concluded that for each of the considered machines, a defect-free forging was obtained for variant 2 preforms. With a lower degree of forging, the material fills the impression well. The developed geometry allows the avoidance of cracks and bends in the shank part of the connecting rod. The results obtained for variant 2 also confirm the appropriate preparation of the billet in the form of cast preforms for the forging process by the application of annealing prior to the process. The defect-free forgings also testify to the correct selection of technological parameters for the forging process.

While investigating the obtained test results for variant 1 preforms with a greater degree of forging, each machine should be considered separately. During the forging process carried out on the screw press, no defect-free forgings were obtained from variant 1 preforms. The application of variant 1 preform on the screw on a screw press with a high deformation rate resulted in the emergence of a defect in the form of cracks in the area where the shank of the rod transitions into the head. Improving the transition radius at the defect’s location in the preform might prove to be the solution to this problem.

Defect-free forgings were formed on the crank press for both variants of the process. It can be assumed that the cast AB-71100 aluminum alloy should be deformed on forging machines with low deformation rates, such as a crank press or hydraulic press.

#### 3.2.2. Quality Test Results for the Connecting Rod Forgings

**Figure 31:**
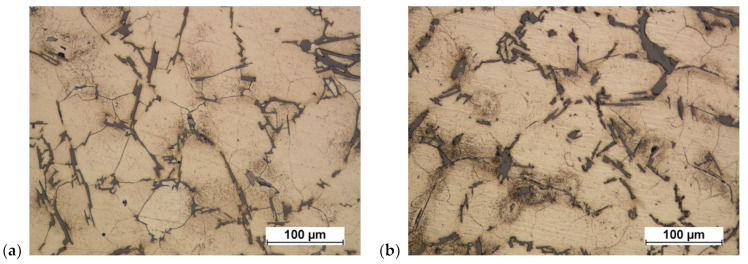
Cast of a connecting rod preform made of AB-71100 aluminum alloy: (**a**) variant 1; (**b**) variant 2.

On the basis of the microstructural tests of cast preforms of connecting rods made of AB-71100 aluminum alloy, the structures shown in Figure 31 were obtained.

AB-71100 aluminum alloy is multi-component. Its microstructure consists of an alpha aluminum solid solution and precipitates of varying natures. There are igneous Al-Si precipitates of eutectic character and slightly thicker intermetallic magnesium-containing phases, e.g., Mg-Si forming Chinese script-type patterns. Most of the zinc is dissolved in a solid solution, and iron forms isolated slightly smaller Al-Fe-Si precipitates. The precipitates are dispersively distributed on the analyzed surfaces.

**Figure 32:**
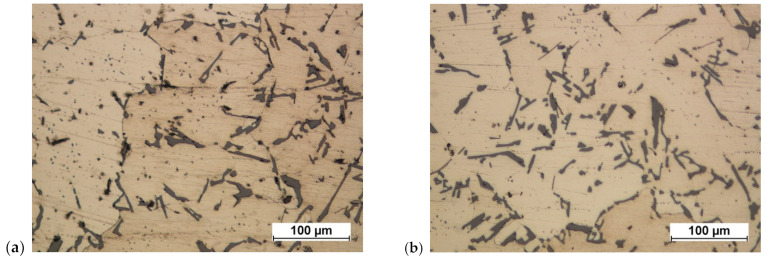
Cast of a connecting rod preform made of AB-71100 aluminum alloy after homogenization: (**a**) variant 1, (**b**) variant 2.

As a result of homogenization, the structure of the aluminum alloy does not undergo significant transformations (Figure 32). Previously invisible grain boundaries appear. The grains themselves are of considerable size. There is little change in the shapes of the discharges. The sharp corners of the coniferous forms become rounded.

**Figure 33:**
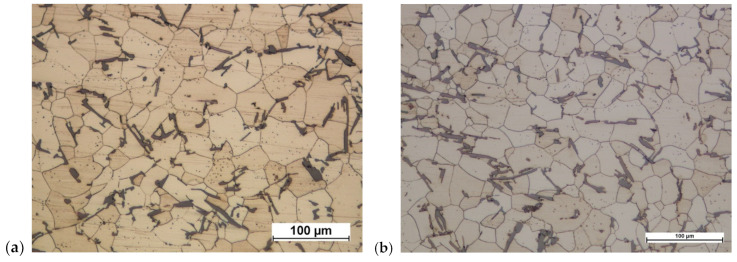
Connecting rod forging forged from a preform in accordance with variant 2: (**a**) on a screw press; (**b**) on a screw press and after heat treatment.

After forging on the screw press, the structure became significantly fragmented. Grain fragmentation and disintegration of parts of particularly larger separations were obtained (Figure 33).

**Figure 34:**
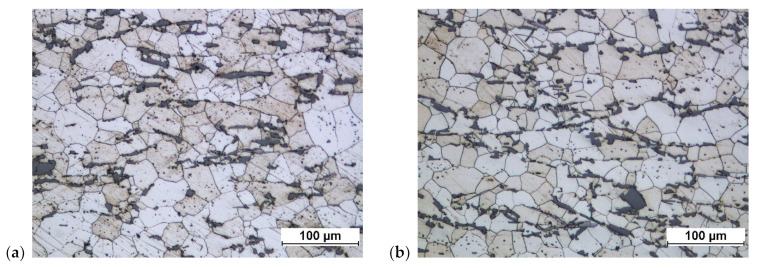
Connecting rod forging forged from a preform in accordance with variant 1: (**a**) on a crank press; (**b**) on a crank press and after heat treatment.

**Figure 35:**
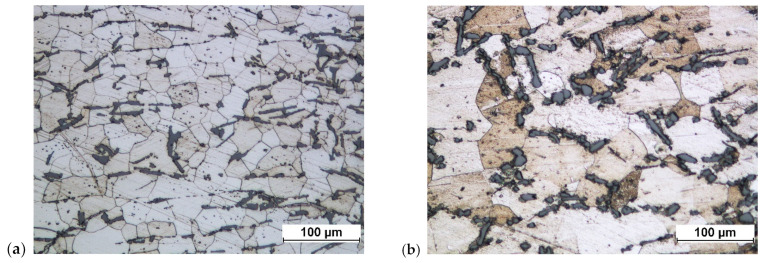
Connecting rod forging forged from a preform in accordance with variant 2: (**a**) on a crank press; (**b**) on a crank press and after heat treatment.

The structure after forging on the crank press is similar to the structure forged on the screw press. After heat treatment, slight grain growth and minor symptoms of the beginning of the spheroidization process of the separations are visible, mainly manifested by the rounding of sharp edges (Figure 34 and Figure 35). Increasing the degree of forging leads to greater grain size reduction.

Table 4 presents the results of hardness measurements for preforms and forgings of connecting rods made of AB-71100 aluminum alloy. After homogenization, a slight decrease in hardness was noted; however, both the forging and heat treatment processes do not significantly change the hardness of the alloy. The homogenization process leads to two parallel phenomena. On the one hand, it is the rounding off of the edges of high-melting precipitates, while on the other hand, some of the phases are dissolved in a solid solution such as Mg_2_Si. The alloy tends to age spontaneously. Therefore, initially, after homogenization, hardness is slightly lower. It returns to the initial level after the full process.

The average values obtained in the tensile strength test are presented in Table 5.

The results of the tensile test indicate that forging improves the plastic properties of the cast AB-71100 aluminum alloy to the greatest extent. However, it does not significantly affect the value of Young’s modulus. The highest values of elongation and tensile strength were obtained for samples after forging on the crank press from variant 1 preform and full heat treatment.

Based on the experimental research in industrial conditions, the following conclusions were formulated:

- Experimental verification in industrial conditions of the novel technology for the forging of connecting rod forgings from preforms cast from AB-71100 aluminum alloy showed that only the geometrical variant 2 of the preform ensures a forging of the assumed shape and dimensions for each machine (screw press, crank press) is obtained;
- The geometrical variant 1 of the preform ensures a forging of the assumed shape and dimensions is obtained only for the forging process carried out on the crank press;
- In order to obtain the correct connecting rod forging from variant 1 preform with a greater degree of forging on the machine with a high deformation rate-screw press, the preform’s geometry should be redesigned on the transition radius between the head and the shank part of the preform;
- The main phenomenon disturbing the process of the forging of connecting rods from preforms cast from AB-71100 aluminum alloy on the screw press includes cracks;
- The results of qualitative studies of the formed forgings indicate favorable changes in the functional properties of the forgings. Although no significant changes in the hardness of the alloy were observed at different stages of the technological process, the results of structural and tensile tests manifest significant differences;
- The structure of the alloy is fragmented as a result of deformation, and AlSi separations are also fragmented;
- The effect of temperature significantly affects the rounding off of crushed fragments of AlSi separations. Both the fragmentation and spheroidization of the separations can favorably influence the improvement of ductile properties;
- Tensile test results indicate improved ductile properties while maintaining high strength-related properties in the form of Young’s modulus and tensile strength;
- Although the studied alloy is mainly used for castings, the application of thermoplastic processes has significantly improved its ductile properties, which may have beneficial effects in selected applications.

## 4. Conclusions

The current study presents new possibilities for manufacturing automotive connecting rods using the innovative technology of die forging on presses from preforms cast from AB-71100 aluminum alloy. Based upon theoretical research and experimental tests, it can be argued that the proposed forming process enables the production of defect-free forgings of appropriate dimensions from preforms cast from AB-71100 alloy. The research also confirmed the possibility of deforming AB-71100 aluminum alloy. The advantages of the proposed technology of connecting rods’ manufacturing over the current production methods entail the following:

- Enhancement of the efficiency and output of the manufacturing process due to shortening the processing time to a single forging operation, in comparison to the multistage forging from a rod and time-consuming mechanical processing of the casting in current operation in the industry;
- Considerable material savings in relation to the multistage forging method in the current application. The technological waste for flash in the standard process amounts to 44.3% of the forging’s mass. In the novel technology of forging from preforms, the waste amounts to 13.5% for variant 2 preform and 18.7% for variant 1 preform;
- Improvement of product quality. A higher product quality is associated with a more favorable structure and high smoothness of the surface. This translates into improved tensile and functional properties when compared with products manufactured by the mechanical processing of castings [39,40,41,42,43,44];
- Possibility of forming connecting rods from less ductile alloys possessing better tensile properties. The use of a shaped, cast preform without metal forming will have a positive effect on the flow kinematics and deformability of the material during forging [45,46,47,48];
- Ecofriendliness of the novel process. It stems from a more eco-friendly production method, i.e., lower waste volume in comparison to the connecting rod manufacturing method in current employment.

The advantage of the proposed technology is its universality of application. The method can be applied to manufacture connecting rods made from other lightweight aluminum alloys.

The numerical analyses conducted in the framework of the study confirmed the advantages of exploiting computer simulations when designing novel technologies [17]. They enable multifaceted analyses to be conducted at the process design stage. In the case of the present study, the simulations enabled the analysis of flow kinematics in the novel process, assessment of the geometry, process analysis concerning stress, strain, cracking and more precise development of experimental tests and reduction in their cost. Based upon the simulations, the final shape of the prototype preforms was determined.

The industrial tests enabled the determination of optimal technological parameters for the proper formation of the connecting rod forging from preforms, verification of the preform geometry on presses with varying deformation rates, and allowed technology demonstrators to be developed and qualitative studies to be conducted.

## 5. Patents

1. Dziubińska A., Winiarski G., Surdacki P., Majerski K., Siemionek E., Szucki M., Method of forging a semi-finished component on a hydraulic press, particularly for the manufacture of a car connecting rod, Patent Office of the Republic of Poland. Patent no. P.431789, Registered 14 November 2019.
2. Winiarski G., Dziubińska A., Surdacki P., Majerski K., Szucki M., Siemionek E., Method of forming a semi-finished product on a hydraulic press, especially for the manufacture of a car connecting rod, Patent Office of the Republic of Poland. Patent no. P.431788, Registered 14 November 2019.
3. Dziubińska A., Winiarski G., Surdacki P., Majerski K., Szucki M., Siemionek E., Method of forming a semi-finished product in a forging device on a hydraulic press, especially for the manufacture of a car connecting rod, Patent Office of the Republic of Poland. Patent no. P.431790, Registered 14 November 2019.
4. Dziubińska A., Winiarski G., Surdacki P., Majerski K., Siemionek E., Szucki M., Method of forging a semi-finished component in a forging device on a hydraulic press, especially for the manufacture of a car connecting rod, Patent Office of the Republic of Poland. Patent no. P.431791, Registered 14 November 2019.

## Figures and Tables

**Figure 4:**
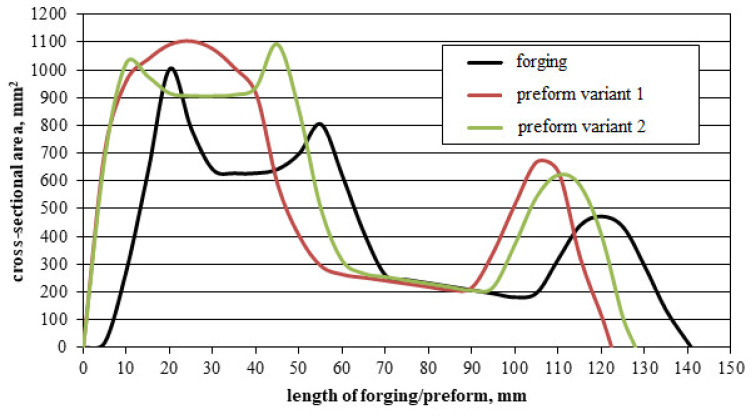
Diagram of cross-sections of forging and preforms.

**Figure 5:**
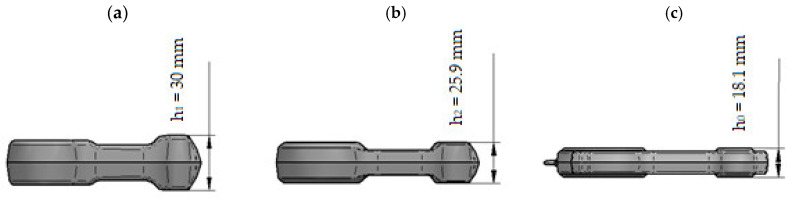
Three-dimensional model of the connecting rod preform: (**a**) variant 1; (**b**) variant 2; (**c**) forging.

**Figure 6:**
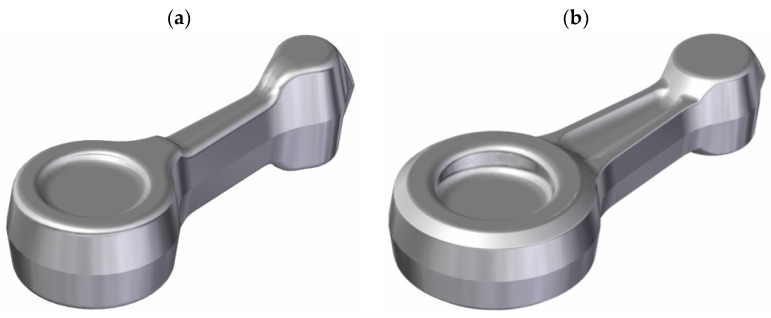
Three-dimensional model of the connecting rod preform: (**a**) variant 1; (**b**) variant 2.

**Figure 7:**
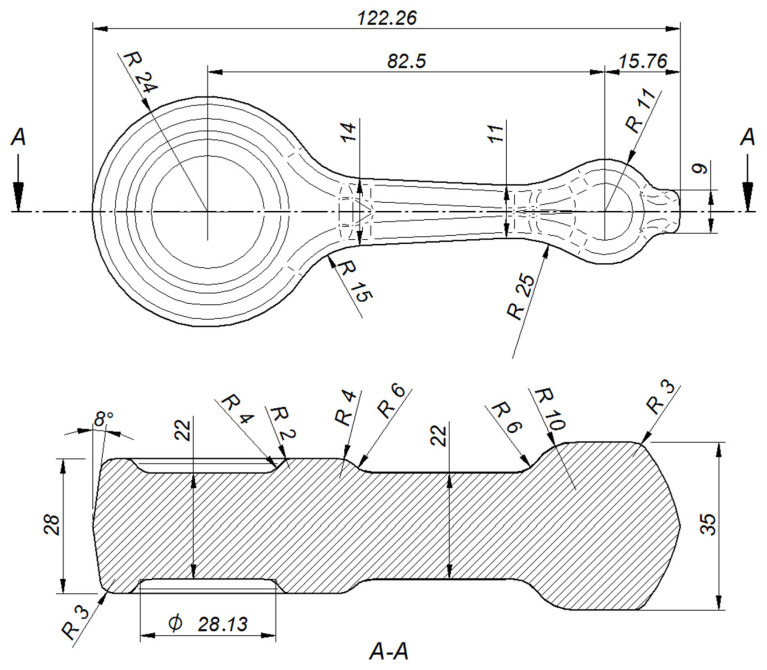
Selected dimensions of the variant 1 preform.

**Figure 8:**
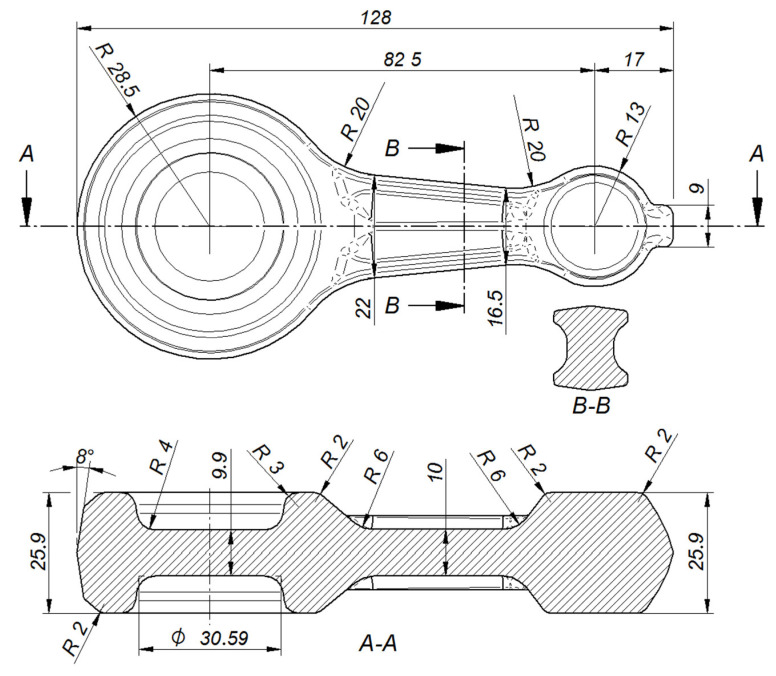
Selected dimensions of the variant 2 preform.

**Table 1:** Nominal chemical composition of EN AB-71100 aluminum alloy used in the experiment (wt%) [28,29].

Al	Zn	Mg	Cu	Fe	Mn	Si	Ti	Other
balance	9–10.5	0.2–0.5	max 0.1	max 0.3	max 0.15	7.5–9.5	max 0.15	0.15

**Table 2:** Heat treatment temperature parameters for cast AB-71100 aluminum alloy [32,33,34,35,36,37].

Type of Heat Treatment	Treatment Conditions
Homogenization	Furnace heating to T = 480 °C, holding at T = 480 °C for 24 h. Then cooling in a furnace to room temperature.
Supersaturation	Not applicable.
Aging	Natural, ambient temperature for 7 days.

**Table 3:** Summary of the volume of geometry for each forging variant and the percentage of process waste for each technology.

Component	Volume of the Component, [mm^3^]	Waste, [%]
variant 1 preform	67,437.2	13.5
variant 2 preform	71,743.6	18.7
bar (current multistage technology)	104,615.0	44.3
forging	58,299.9	-

**Table 4:** Results of hardness measurements of the connecting rod preforms and forgings made of AB-71100 alloy.

Sample Number	State of the Sample	Mean of Measurements [HV05]	Standard Deviation
1.	Preform casting variant 1	107.9	3.9
2.	Preform casting variant 2	103.8	3.8
3.	Preform casting variant 1 after homogenization	101.5	1.4
4.	Preform casting variant 2 after homogenization	101.2	2.4
5.	Forging forged on the screw press from a preform casting according to variant 2	102.4	2.8
6.	Forging forged on the screw press from a preform casting according to variant 2 after heat treatment	107.2	5.4
7.	Forging forged on the crank press from a preform casting according to variant 1	102.1	3.1
8.	Forging forged on the crank press from a preform casting according to variant 1 after heat treatment	103.4	1.2
9.	Forging forged on the crank press from a preform casting according to variant 2	102.0	2.0
10.	Forging forged on the crank press from a preform casting according to variant 2 after heat treatment	103.2	2.6

**Table 5:** Results of the static tensile test of samples made of preforms and forgings of connecting rods made of AB-71100 alloy.

State of the Sample	Tensile Strength Rm [MPa]	Young’s Modulus E [GPa]	Yield Strength R_02_ [MPa]	Elongation A_5_ [%]
Preform casting variant 1 after heat treatment (supersaturation + aging)	243.9	71.8	193.7	3.3
Preform casting variant 2 after heat treatment (supersaturation + aging)	237.8	70.5	191.1	3.2
Forging forged on a screw press from a preform casting according to variant 2 after heat treatment (supersaturation + aging)	246.4	72	182.6	5.2
Forging forged on a crank press from a preform casting according to variant 1 (supersaturation + aging)	276.8	71.2	208.3	6.2
Forging forged on a crank press from a preform casting according to variant 2 (supersaturation + aging)	240.6	71.6	196.2	5.7

## Data Availability

Data are contained within the article.
